# Brain structural alterations associated with impulsiveness in male violent patients with schizophrenia

**DOI:** 10.1186/s12888-024-05721-3

**Published:** 2024-04-15

**Authors:** Juntao Lu, Ningzhi Gou, Qiaoling Sun, Ying Huang, Huijuan Guo, Dian Han, Jiansong Zhou, Xiaoping Wang

**Affiliations:** 1 Department of Psychiatry, National Clinical Research Center for Mental Disorders, and National Center for Mental Disorders, The Second Xiangya Hospital of Central South University, Changsha, Hunan 410011 China; 2 Department of Psychiatry, the First Affiliated Hospital, Medical College of Xi ’an Jiaotong University, Xi’an, Shaanxi 710061 China

**Keywords:** Schizophrenia, Violent behavior, MRI, Impulsive, Volume

## Abstract

**Background:**

Violence in schizophrenia (SCZ) is a phenomenon associated with neurobiological factors. However, the neural mechanisms of violence in patients with SCZ are not yet sufficiently understood. Thus, this study aimed to explore the structural changes associated with the high risk of violence and its association with impulsiveness in patients with SCZ to reveal the possible neurobiological basis.

**Method:**

The voxel-based morphometry approach and whole-brain analyses were used to measure the alteration of gray matter volume (GMV) for 45 schizophrenia patients with violence (VSC), 45 schizophrenia patients without violence (NSC), and 53 healthy controls (HC). Correlation analyses were used to examine the association of impulsiveness and brain regions associated with violence.

**Results:**

The results demonstrated reduced GMV in the right insula within the VSC group compared with the NSC group, and decreased GMV in the right temporal pole and left orbital part of superior frontal gyrus only in the VSC group compared to the HC group. Spearman correlation analyses further revealed a positive correlation between impulsiveness and GMV of the left superior temporal gyrus, bilateral insula and left medial orbital part of the superior frontal gyrus in the VSC group.

**Conclusion:**

Our findings have provided further evidence for structural alterations in patients with SCZ who had engaged in severe violence, as well as the relationship between the specific brain alterations and impulsiveness. This work provides neural biomarkers and improves our insight into the neural underpinnings of violence in patients with SCZ.

## Background

Evidence suggests that patients with schizophrenia (SCZ) are at a significantly higher risk of violent behavior compared with the general population [1]. A meta-analysis indicated that the odds ratio (OR) for the comparison of violent behaviors between men with SCZ and healthy controls (HC) was 4.5 (95% CI, 3.6–5.6) [2]. Violent behaviors in SCZ may lead to serious harm to others, higher healthcare costs, and increased stigma among the patients [3]. Therefore, it is vital to work out the underpinnings of violence in SCZ. Violence is associated with numerous sociodemographic and clinical factors [1], such as young age, male sex, economic deprivation, psychotic symptoms, impulsivity, and comorbidity with substance abuse or personality disorder [4, 5]. An increasing number of studies have implicated the importance of neurobiological factors in violent behaviors, suggesting that neurobiological and psychosocial factors may be intricately intertwined with violence in SCZ. However, the etiology basis of violent behavior in SCZ is still not sufficiently understood by now, necessitating the exploration of the neural basis of violent behaviors in individuals with SCZ, as this may help us find evidence-based approaches to reduce the risks of violence in SCZ.

Structural magnetic resonance imaging (sMRI) is a non-invasive and high-resolution imaging technique commonly used in neurobiological studies. Recent sMRI studies have indicated that violent behaviors in patients with schizophrenia are linked to alterations in multiple cerebral regions, including the frontal and temporal lobes [6–8], limbic system [9, 10], and other regions such as the cerebellum [11]. Yu et al. found that patients with SCZ who had engaged in violence showed significantly reduced volume in the left frontal pole and reduced thickness in the right inferior parietal gyri, as compared with patients without any history of violence [12]. Another study exhibited deficits of gray matter volume (GMV) in the inferior and middle temporal gyri, temporal pole, fusiform gyrus, and insula in schizophrenia patients with violence (VSC) compared to schizophrenia patients without violence (NSC) [13]. An association has also been found between hostility and the GMV of the left inferior temporal cortex in patients with VSC [8]. Volume alterations in the limbic system, especially in the hippocampus and amygdala, are also reported in some studies [14, 15]. A study reported decreased GMV of the hypothalamus in patients with VSC, compared to NSC and HC [16], and the GMV of the hypothalamus was found negatively correlated with the scores of PANSS and MOAS. Furthermore, reduced bilateral hippocampal GMV was also found in patients with SCZ who committed homicide, as compared to those with NSC and HC [17]. Some other researchers found that patients with VSC had reduced GMV in the bilateral cerebellum, BA 39/40 [11], putamen, left cuneus/precuneus and parietal cortex [18], as well as decreased cortical thickness in sensorimotor regions [19], compared to those with NSC. However, some neuroimaging studies did not find any significant difference in the brain structure between the VSC and NSC [7, 15, 20, 21]. Due to the disparities in current findings, the structural changes associated with violence in SCZ are still unclear, nor is its association with clinical risk factors in SCZ, which indicated the necessity to explore the neurobiological underpinning of violent behavior in individuals with SCZ.

Impulsivity is a predisposition toward rapid and unplanned reactions to stimuli without regard to the negative consequences [22]; it is also a crucial risk factor for violence in patients with SCZ. A strong association between impulsiveness and a higher risk of violence in patients with psychosis has been found in a meta-analysis [23]. However, brain morphological brain correlates of impulsivity in violent patients with SCZ have been poorly investigated. Kumari et al. found a possible association between impulsivity and reduced hippocampal volumes in VSC [9], Hoptman et al. reported that a reduced cortical thickness in ventral prefrontal regions was correlated with higher impulsivity scores [24], and according to Baumann et al., impulsivity was found to be positively correlated with the frontal cortical thickness in patients with psychosis [25]. Another study demonstrated that impulsivity might be mediated by limbic brain structures and controlled by frontotemporal brain regions [26]. The above findings implicate that impulsivity might be associated with brain regions involved in emotion processing and control, but the specific regions involved were inconsistent.

The disparities and even contradictory results might be attributed to the relatively small sample size, the inconsistent definitions of violence, and the heterogeneity of the studied patient groups [8, 27]. For instance, in some studies, violence was defined as physical harm to others, with or without homicide, while in some other studies, violence was defined only using the score of some scales [12, 13, 16, 17]. However, the examination of neurobiological underpinnings of violence that has caused serious consequences can be more helpful. In addition, the results of a study can also be affected by comorbidities, such as personality disorders and substance addiction, which are also found to be associated with violence [28, 29] and possibly associated with alterations of brain structure [18]. Furthermore, numerous studies control the level of severity of psychiatric symptoms, but few of them conducted subgroup analysis on the positive and negative symptoms separately. Studies found that positive symptoms were often associated with an increased risk of violence [30] as well as a lower GMV of the fronto-temporo-parietal regions [31, 32], while negative symptoms were often associated with altered volume of some brain structures such as the putamen [33]. Thus, controlling for positive and negative symptoms separately may also be necessary for studies of violence in SCZ.

Based on the above evidence, the violent participants in the present study were recruited form the forensic psychiatry department rather than general wards to ensure a documented history of violent behavior. In addition, we excluded confounding factors that were frequently overlooked in previous studies. The present study aimed to explore the morphometric characteristics associated with violence in SCZ and to evaluate the association between brain structure alterations and impulsiveness using a voxel-based morphometry approach and whole-brain analyses. We hypothesized that patients with a history of violence might show structural alterations in the frontotemporal and limbic regions, which are associated with emotion processing and control, and that the alterations might be correlated with impulsiveness, thus contributing to a higher risk for violent behavior in SCZ.

## Methods

### Participants

The study procedures were approved by the Ethics Committee of the Second Xiangya Hospital. We recruited 45 patients with schizophrenia who had engaged in serious violent behaviors and referred to the forensic psychiatry department of the Second Xiangya Hospital (China) for examination. According to previous study [16], the present research defined violence as behaviors causing severe physical injuries to others, including homicide and other serious assaults. What’s more, for better quantitative description, we also required that the scores of the Modified Overt Aggression Scale (MOAS) to be more than 4 in the group of VSC [8, 34]. The inclusion criteria of VSC were: (1) aged between 18 and 65 years; (2) male and right-handed; (3) suitability for magnetic resonance imaging (MRI) scan with provided written informed consent; (4) diagnosed schizophrenia by two experienced psychiatrists using the International Classification of Diseases Version 10 (ICD-10); (5) with scores of MOAS higher than 4. A total of 45 age and symptom matched patients, with no history of violence, were recruited from the psychiatry wards of the same hospital. These participants adhered to the same inclusion criteria, with the only exception being the absence of aggressive behavior towards people or objects. Exclusion criteria were (1) with a history of substance abuse or dependence; (2) diagnosed with other psychiatric disorders; (3) with a history of severe head injury that caused a loss of consciousness. During the same period, we also recruited 53 age-matched healthy individuals (i.e., HC) who met the all the inclusion criteria and none of the exclusion criteria, except that none of them had a diagnosis of schizophrenia.

### Clinical assessments and socio-demographic information

A self-designed standardized form was used to record the socio-demographic information, including age, duration of illness, marital status, age of onset, and dose equivalents of antipsychotics based on defined daily doses (DDDs) [35].

Psychotic symptoms were assessed using the Positive and Negative Syndrome Scale (PANSS) [36]. The PANSS consists of 30 items, and each item is rated on a Likert scale from 1 to 7 (i.e., from asymptomatic to extremely symptomatic). The items in PANSS are grouped into five factors, i.e., disorganization, excitement, depression, positive symptoms, and negative symptoms [37]. The factor of excitement, including P4 (Excitement), P7 (Hostility), G8 (Uncooperativeness) and G14 (Poor impulse control), is often used to reflect aggressive behaviors [38]. The item of G14 was used to measure the level of impulsiveness.

The acts of violence were evaluated using the MOAS. The MOAS comprises of four domains, i.e., verbal aggression, aggression towards objects, auto-aggression, and physical aggression, which has been validated in the Chinese population [39].

### Acquisition and processing of neuroimaging data

All the structural MRI brain images were acquired using a 3.0 T scanner (Philips Medical Systems). The parameters of the high spatial resolution T1-weighted sequence were: repetition time (TR) 8.2 ms, echo time (TE) 3.8 ms, 188 slices, field of view (FOV) 256 × 256 mm, date matrix 256 × 256, voxel size 1 × 1 × 1 mm.

For data analyses, the CAT12 toolbox (https://neuro-jena.github.io/cat//index.html) and the SPM12 software (https://www.fil.ion.ucl.ac.uk/spm/software/spm12/) in Matlab 2021a were used. Structural images were processed with the following steps: (1) excluding images which artifacts or gross anatomical abnormalities; (2) segmenting the anatomical images into the cerebrospinal fluid (CSF), gray matter (GM) and white matter (WM); (3) the GM images were normalized to Montreal Neurological Institute (MNI) space using the Diffeomorphic Anatomical Registration Through Exponentiated Lie Algebra (DARTEL) [40]; (4) statistical quality control using the function of “checking sample homogeneity” in the CAT12 toolbox; and (5) smoothing the GM images with a Gaussian Kernel of 8 mm.

### Statistics

The demographic and clinical characteristics were analyzed using Statistical Package for Social Sciences Version 25.0 with the level for significance of effect set at *P* = 0.05 (two-tailed). The inter-group differences of continuous variables were analyzed using the independent samples t-tests or one-way analysis of variance (ANOVA) as appropriate. Group comparisons for non-normally distributed data were assessed with the non-parametric Mann-Whitney U test. The Chi-square (***χ***^***2***^) test was performed to assess the differences of categorical variables.

All the statistical analyses of volumetric imaging data were performed using the SPM12 statistical module. The whole-brain voxel level comparison of GMV was analyzed using one-way ANCOVA between the VSC, NSC and HC groups with the level of education, age and total intracranial volume (TIV) as covariates. The significant clusters identified in the ANCOVA analysis were extracted as regions of interest (ROI) masks for post-hoc t test to reveal GMV differences between any two groups. The DDDs, positive and negative symptom scores, and duration of illness were used as covariates in the post-hoc t test between VSC and NSC. The significance level was set at *P* < 0.05 correction at the voxel level using familywise error (FWE) for multiple comparisons. The Spearman’s correlation was used to identify the correlation between significant brain regions and impulsiveness (indicated by the PANSS score).

## Results

### Comparisons of demographic and clinical statistics

No significant difference in age (*P* = 0.06) was noted among VSC, NSC and HC groups as presented in Table 1. Inter-group differences were found in levels of education (*P*<0.001), with HC and NSC having achieved higher years of education compared to the VSC. Significant differences were also found in marital status between the three groups, with more patients being single in the VSC and NSC groups, as compared to the HC. No significant difference was found in the duration of illness (*T* = 1.496, *P* = 0.138), hospitalization history (***χ***^***2***^ = 0.169, *P* = 0.280), and age at onset (*T* = 0.463, *P* = 0.644) between the two patient groups. As expected, the VSC group had a significantly higher score of MOAS than the NSC and HC groups (*F* = 324.19, *P* < 0.001). The VSC group also had a significantly higher level of excitement and impulsiveness indicated by PANSS compared to the NSC group, while no significant differences were identified in scores of positive symptoms, negative symptoms, disorganization and depression between the VSC and NSC groups. No significant difference was found in DDDs between VSC and NSC groups, and olanzapine was the primary treatment for the majority of patients during the study.

**Table 1:** Demographic and clinical characteristics of the three groups

	VSC (*N* = 45)	NSC (*N* = 45)	HC (*N* = 53)	F/χ^2^/T	P
Age, years (mean ± SD)	30.82 ± 7.94	27.93 ± 6.33	31.17 ± 7.14	2.872	0.060
Sex (M/F)	45/0	45/0	53/0		
Education, years (mean ± SD)	10.22 ± 2.53	12.44 ± 3.08	12.66 ± 3.08	9.985	**< 0.001**
**Marital status (n,%)**					
Single	36 (80.0)	39 (86.7)	27 (71.3)	22.481	**< 0.001**
Married	5 (11.1)	4 (8.9)	23 (22.4)		
Divorced	4 (8.9)	2 (4.4)	3 (6.3)		
DDDs, mg (mean ± SD)	6.77 ± 7.74	10.18 ± 8.80	-	-1.948	0.055
Duration of illness, months (mean ± SD)	75.36 ± 59.19	57.09 ± 56.71	-	1.496	0.138
Age of onset, years (mean ± SD)	23.59 ± 6.74	22.90 ± 6.99	-	0.463	0.644
**Hospitalization history (n,%)**				1.169	0.280
Yes	15 (33.3)	20 (44.4)	-		
No	30 (66.7)	25 (55.6)	-		
**On treatment (n,%)**			-	1.813	0.178
Yes	34 (75.6)	39 (86.7)	-	2.500	0.114
No	11 (24.4)	3 (13.3)	-		
MOAS score	21.27 ± 5.56	2.92 ± 4.25	0.11 ± 0.31	324.19	**< 0.001**
**PANSS score**					
Positive	14.98 ± 4.78	14.73 ± 3.43	-	0.279	0.781
Negative	17.44 ± 6.90	14.93 ± 5.94	-	1.85	0.068
Disorganization	6.58 ± 3.58	7.11 ± 3.23	-	-0.742	0.469
Depression	4.13 ± 1.58	4.96 ± 2.37	-	-1.936	0.056
Excitement	12.07 ± 4.60	6.76 ± 2.79	-	6.618	**< 0.001**
Impulsiveness	5.04 ± 2.41	1.78 ± 1.13		8.233	**< 0.001**

VSC, schizophrenia patients with violence; NSC, schizophrenia patients without violence; HC, healthy controls; MOAS, Modified Overt Aggression Scale; PANSS, Positive and Negative Syndrome Scale

The bold values means that the values are followed by their respective classfications

### Volumetric analyses

**Fig. 1:**
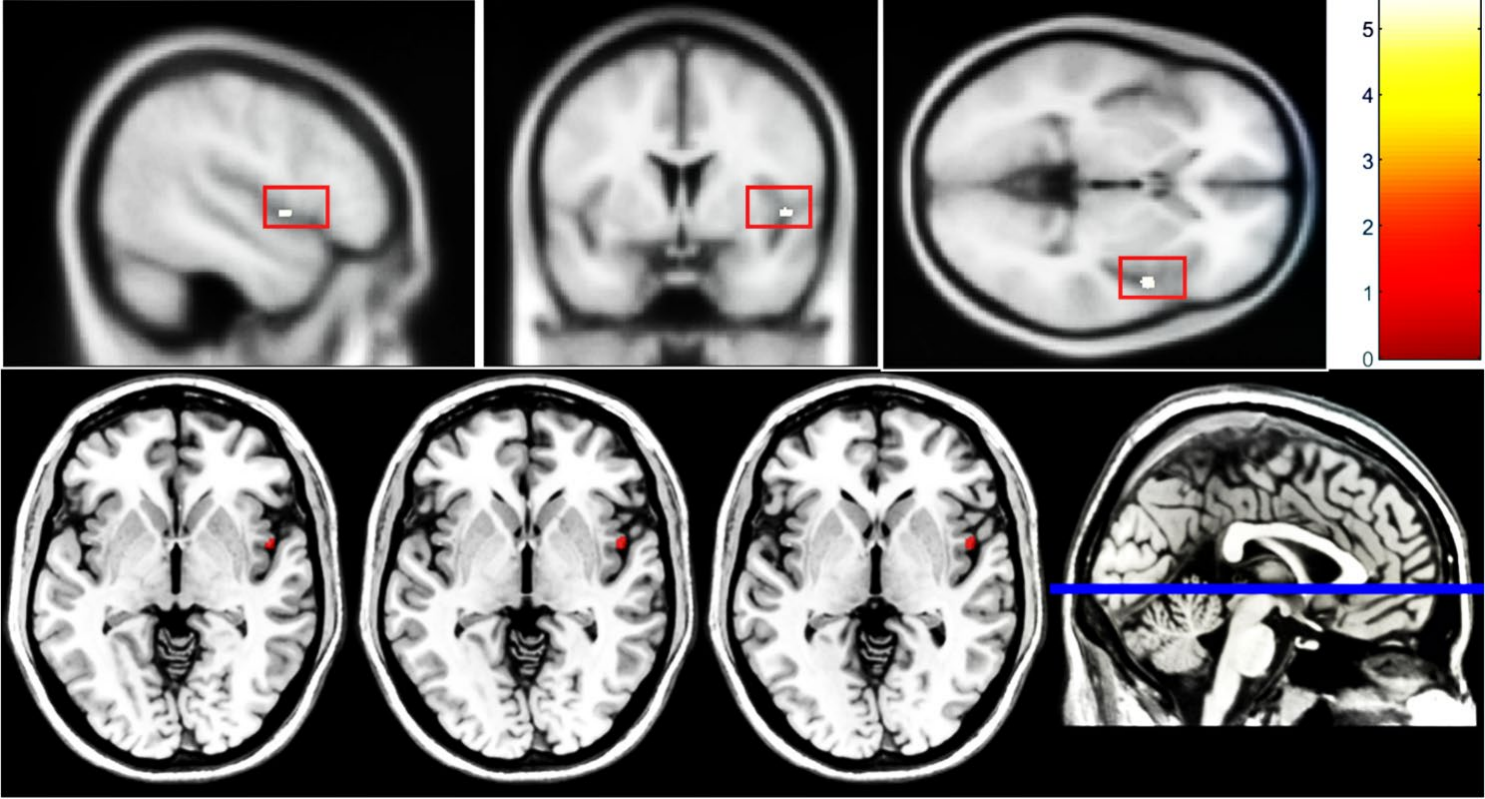
Reduction in GMV of the right insula in the VSC group compared to the NSC group (Peak voxel significant at *p* < 0.05, voxel-level familywise error (FWE) correction)

**Fig. 2:**
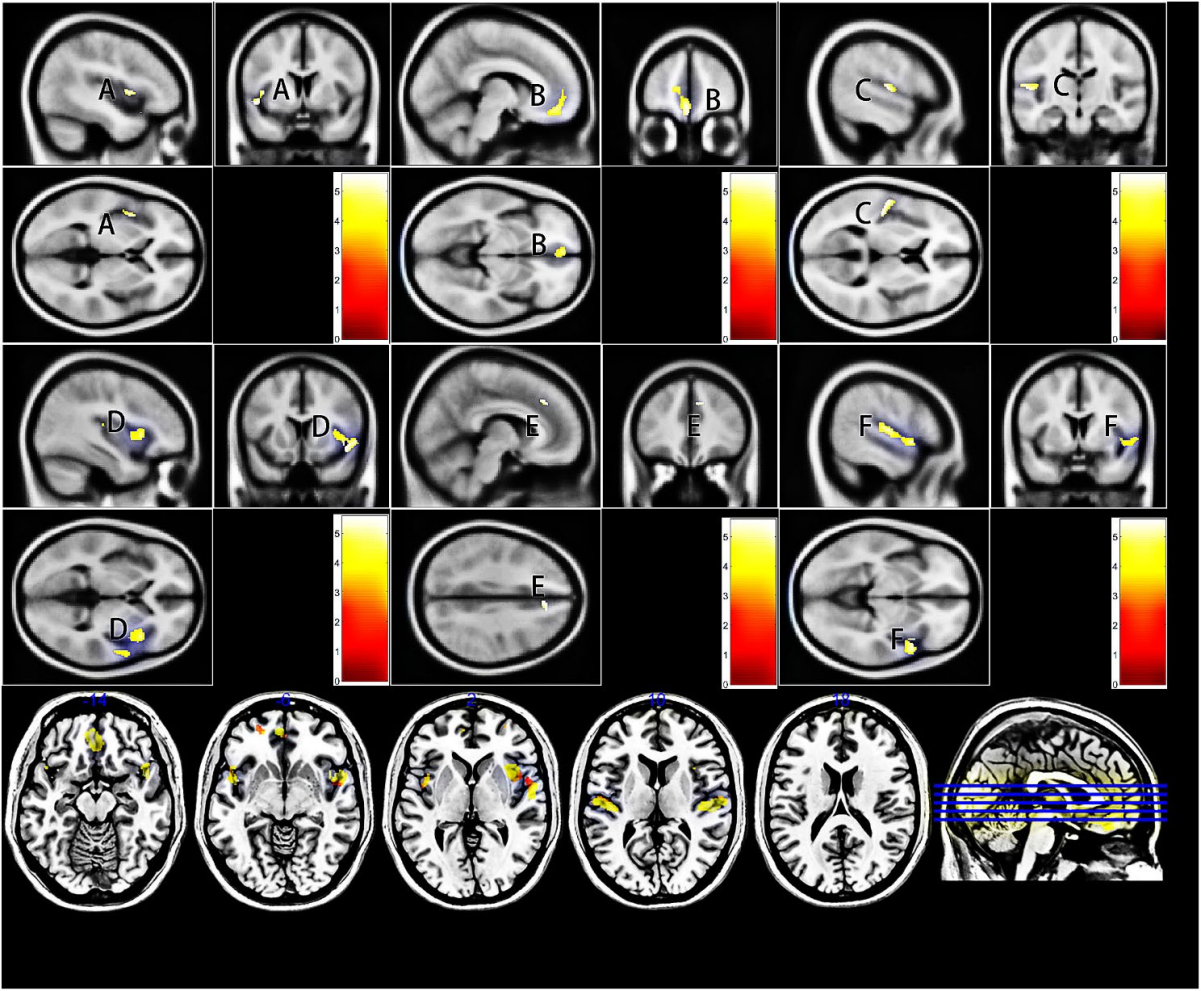
Decrease of GMV in the VSC group compared to the HC group (Peak voxel significant at *p* < 0.05, voxel-level familywise error (FWE) correction). (**A**) left superior temporal gyrus; (**B**) left insula; (**C**) left superior frontal gyrus, medial orbital; (**D**) right insula; (**E**) right superior frontal gyrus, medial; (**F**) right superior temporal gyrus; (**G**) left superior frontal gyrus, orbital part; (**H**) right temporal pole: middle temporal gyrus

**Fig. 3:**
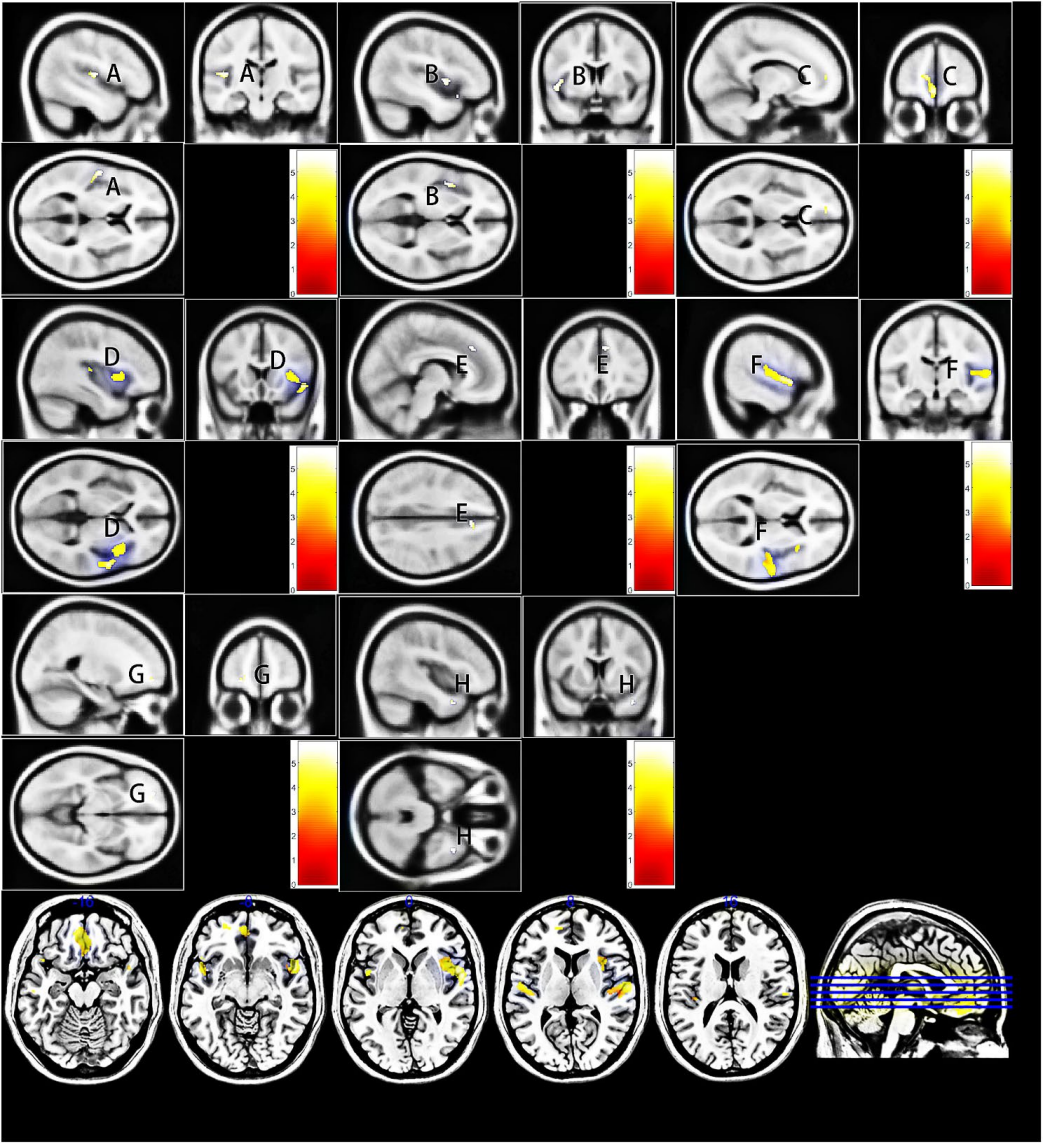
Decrease of GMV in the NSC group compared to the HC group (Peak voxel significant at *p* < 0.05, voxel-level familywise error (FWE) correction). (**A**) left insula; (**B**) left superior frontal gyrus, medial; (**C**) left superior temporal gyrus; (**D**) right insula; (**E**) right superior frontal gyrus, medial; (**F**) right superior temporal gyrus

After controlling for age, level of education and total intracranial volume of the whole brain, significant differences in GMV were found by one-way ANCOVA in a variety of regions between three groups, including the right temporal pole: middle temporal gyrus (TPOmid), bilateral superior temporal gyrus (STG), left superior frontal gyrus, medial orbital (ORBsupmed), left superior frontal gyrus, orbital part (ORBsup), right superior frontal gyrus, medial (SFGmed), and left gyrus rectus (REC) and bilateral insula (INS). Post-hoc t test showed reduced GMV of right INS in the VSC group compared with NSC group (Fig. 1) and right TPOmid and left ORBsup in the VSC group compared to the HC group (Fig. 2). Furthermore, regions with decreased GMV shared by the VSC and NSC groups involved the bilateral STG, left ORBsupmed, right SFGmed and bilateral INS, as compared with the HC (Figs. 2, 3; Table 2). No brain regions were identified with a higher GMV in the VSC or NSC group as compared with the HC.

**Table 2:** Significant differences in GMV between the three groups

Contrast	Hemisphere	Brain region	MNI coordinates			Cluster size	T	P
			x	y	z			
VSC > NSC	right	insula	48	1.5	-1.5	23	3.84	0.023
HC > NSC								
	left	Superior frontal gyrus, medial orbital	-3	46.5	-13.5	1145	5.04	< 0.001
		Gyrus rectus						
	right	Insula	46.5	16.5	-6	2015	5.56	< 0.001
		Superior temporal gyrus						
	left	Insula	-46.5	16.5	-6	290	4.51	0.003
	left	Superior temporal gyrus	-43.5	-21	9	376	4.86	0.001
	right	Superior frontal gyrus, medial	3	42	34.5	104	4.77	0.001
HC > VSC								
	right	Temporal pole: middle temporal gyrus	39	10.5	-31.5	30	5.58	< 0.001
	left	Superior frontal gyrus, orbital part	-19.5	51	-6	66	5.54	< 0.001
	left	Superior frontal gyrus, medial orbital	-1.5	43.5	-15	1161	6.35	< 0.001
		Gyrus rectus						
	right	Insula	52.5	9	-4.5	2287	7.37	< 0.001
		Superior temporal gyrus						
	left	Insula	-49.5	16.5	-6	336	5.87	< 0.001
	left	Superior temporal gyrus	-45	-18	10.5	392	5.54	< 0.001
	right	Superior frontal gyrus, medial	0	40.5	30	104	5.24	< 0.001

NSC, schizophrenia patients without violence; VSC, schizophrenia patients with violence; HC, healthy controls; corrected for multiple comparisons after Family Wise Error (FWE), peak voxel significant at *p* < 0.05

### Correlation analysis

**Fig. 4:**
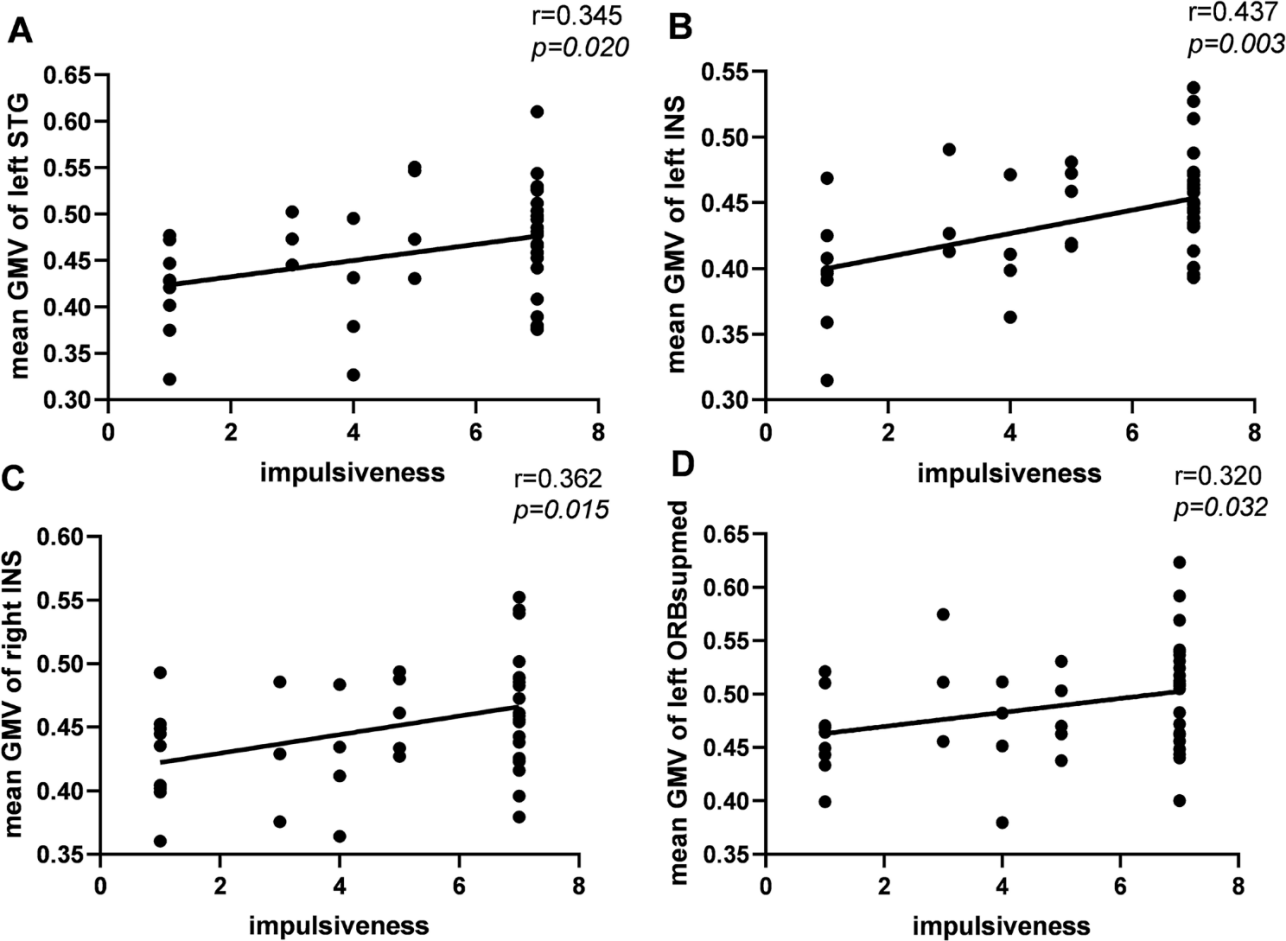
Spearman correlation analysis between the mean GMV of the significant region and the impulsiveness score in the VSC group. (**A**): left superior temporal gyrus (STG), *r* = 0.345, *P* = 0.020; (**B**): left insula (INS), *r* = 0.485, *P* = 0.001; (**C**): right insula (INS), *r* = 0.388, *P* = 0.008; (**D**): left superior frontal gyrus, medial orbital part (ORBsupmed), *r* = 0.314, *P* = 0.036

As shown in Fig. 4, impulsiveness exhibited significantly positive correlation with the GMV in the bilateral INS, the left STG, and the left ORBsup in the VSC group. No significant correlation was found between impulsiveness and GMV alteration in the NSC group.

## Discussion

In the present study, the VSC showed reduced GMV of the right INS compared with the NSC, and a decline of GMV was found in a variety of areas involving the frontal, temporal lobe and the limbic system in patients with VSC compared with the HC; specifically, these areas included the right TPOmid, left ORBsup, right SFGmed, left ORBsupmed, bilateral STG, bilateral INS, and left REC. Compared with the HC, the NSC group also showed reduced GMV of the left REC, left ORBsupmed, bilateral STG, bilateral INS, and right SFGmed. Our findings suggested that a more pronounced reduction of gray matter volume in the frontal and temporal lobes was present in patients with VSC compared to those with NSC, indicating that VSC might be associated with more volumetric deficits. The volume alteration in regions of VSC appeared to be associated with impulsiveness, indicating that poor impulse control might play an important role in the neurobiological basis of violence in SCZ. Compared to prior studies, the whole-brain approach was superior to the analysis of specific isolated regions in the identification of regions associated with violence, as the analysis of isolated regions could be insufficient to reveal the neurobiological underpinnings due to the complex phenotypes of violence [7, 41]. In addition, all the participants with VSC in the present study were free of substance abuse and personality disorder, thus, it is reasonable to infer that the brain structural alterations of these participants might be related to violence [13].

In our study, the VSC exhibited a significant decline in right INS compared to the NSC. Previous research has consistently highlighted the association between the INS and violent behavior [42]. For instance, Nummenmaa et al. observed lower gray matter density in the INS among violent offenders with psychopathic traits [43], while Tiihonena et al. identified reduced volume in the right INS among violent offenders compared to healthy controls [44]. The INS serves as a key hub within the emotional salience network [45], and believed to play an important role in the discrimination between self-generated and external information [46] and to be associated with the feeling of disgust [47]. Dysfunction in this brain area might be linked to a lack of inhibition towards harming others. Consistent with our study, Kuroki et al. also found deficits in the INS among violent patients with schizophrenia [48]. The present study also identified a positive association between INS and impulsiveness. A prior study demonstrated that greater cortical thickness in the INS is linked to higher levels of impulsivity and riskier choices in healthy individuals [49]. Our findings bridged the relationship between impulsiveness and GMV of INS. Patients with a mild deficit in the INS might lean towards engaging in impulsive aggression, while others may be predisposed to non-emotional violence, such as premeditated violence [50–52]. Additional studies found the volume of INS associated with affective empathy [53] and callous-unemotional traits [53, 54], supporting the association between impairments of the INS-related emotion process and violence types in SCZ. In addition, the volume reduction of INS was observed in both the VSC and NSC compared with HC, and might be associated with positive and negative symptoms [32, 55], suggesting that INS abnormalities might also be correlated with the disease itself [56]. This study provides primary evidence that the deficits in GMV of INS play a crucial role in both violence and impulsivity among patients with schizophrenia.

We found brain structural deficits in the temporal lobe, including the right TPOmid and bilateral STG in VSC compared to the HC, while NSC only showed decreased GMV in bilateral STG. In line with our present findings, several prior studies have reported significant differences in the regions at the temporal pole (TP) in violent individuals with schizophrenia. For instance, a significantly reduced regional volume of the temporal pole and other temporal regions including the inferior and middle temporal gyri was revealed in VSC compared to NSC [13]. Structural deficits in the temporal lobe were also found in individuals with psychopathy or antisocial behavioral problems [57, 58]. For instance, Bertsch et al. found a decreased GMV of TP in violent patients with borderline personality disorder compared with healthy individuals [59]. The TP is the most rostral part of the temporal lobe, which is involved in the modulation of the ventral stream [60] and is considered to be associated with face processing [61], and processing of emotional cues associated with stimuli of various modalities [62, 63]. Thus, the GMV deficits of TP might be related to a higher risk for violent behavior in SCZ by interfering with the integration of information regarding facial and emotional processing. The present study further highlighted the important role of the TP in violent patients with SCZ.

In addition to the temporal lobe, a significant decline of GMV was also found in the ORBsup in patients with VSC, but not in patients with NSC. In line with our findings, Narayan et al. also found reduced cortical thickness in the orbitofrontal cortex (OFC) in violent patients with antisocial personality disorder [19]. The OFC plays an important role in emotion regulation, information processing, decision-making and learning [64, 65]. Davidson et al. indicated that dysfunction in the neural system including OFC, which controls affective regulation, might be associated with aggression [66]. These results indicated that deficits in ORBsup might be associated with violence due to deficits in emotion regulation and information processing. Moreover, we also found a positive association between impulsiveness and the GMV of ORBsupmed in patients with VSC. Similar to our findings, Hoptman et al. found that an increase in the volume of the white and gray matter of OFC was positively associated with a higher level of aggression in patients with SCZ [67]. The decreased GMV of ORBsupmed was observed in the VSC groups, indicating the positive correlation might result from different subtypes of violence. The impulsive violence and other subtypes of violence might have different neural circuits, suggesting the patients with a mild impairment in ORBsupmed are more likely to present with a higher level of impulsiveness.

In the present study, a reduction in GMV was observed in the bilateral STG in both the NSC and VSC groups, compared with HC. Smaller volume of bilateral STG, which associated with severity of auditory verbal hallucinations and delusions [32, 68], was also found in NSC and VSC compared with HC, suggesting that the decreased GMV in these areas might be specific to the disease per se [69]. The positive correlation between the GMV of STG and impulsiveness was also found in the present study. STG has been found to be involved with emotional processing and executive cognitive function [70, 71]. A previous study found that impulsive decision-making was associated with morphological alterations in the superior temporal area [72]. A current study on meta-regression revealed a positive relationship between impulsivity and risky decision-making [73], suggesting that the association between impulsiveness and STG might be explained by the function of executive cognitive and emotional processing of this region.

It is noteworthy that the alterations in brain areas associated with violence found in the present study partly overlap with abnormalities observed in personality disorders, such as GMV of the temporal lobe and INS [74–76]. The diagnosis of personality disorder has been excluded in this study, suggesting a potential shared neurobiological basis for violence in patients with SCZ and individuals with personality disorders.

The present study also has several limitations. First, the sample size in this study was limited. A small sample size might yield a variety of results that are challenging to replicate [77, 78]. Consequently, the findings of our study might be considered as preliminary work, necessitating follow-up investigations with significantly larger samples. Second, the analysis of impulsiveness was based on a single item of PANSS, while the impulsiveness is a complex construct. Therefore, our future studies should employ more reliable measurements to assess impulsiveness. Third, the level of education was significantly different between the three groups. Although education was used as a covariate, the influence of this factor might not be fully eliminated.

## Conclusions

The present study has identified some structural differences between patients with schizophrenia who had a history of violent behavior and those who did not engage in violence. Our study found reduced GMV of INS is associated with violence in patients with schizophrenia, and structural abnormalities in TP and ORBsup in patients with a history of violence rather than those without such a history, which might provide a basis for the generalizability of structural deficits to a larger patient group associated with violence and schizophrenia. In addition, the GMV alteration of ORBsupmed, INS and STG were found to be positively correlated with impulsiveness in VSC, indicating that impulsiveness might be associated with particular alterations of brain structure. Future studies on the morphometric characteristics associated with violence in SCZ may help to examine whether the structural alteration found in the present study can be used as neural biomarkers to predict future violence in patients.

## Data Availability

Due to ethical restrictions and personal data protection, data are only available from the corresponding author upon reasonable request.

## Abbreviations

SCZ: schizophrenia
GMV: gray matter volume
VSC: schizophrenia patients with violence
NSC: schizophrenia patients without violence
HC: healthy controls
sMRI: structural magnetic resonance imaging
PANSS: Positive and Negative Syndrome Scale
MOAS: Modified Overt Aggression Scale
DDDs: defined daily doses
ANOVA: one-way analysis of variance
TPOmid: temporal pole: middle temporal gyrus
STG: superior temporal gyrus
ORBsupmed: superior frontal gyrus, medial orbital
ORBsup: superior frontal gyrus, orbital part
SFGmed: superior frontal gyrus, medial
REC: gyrus rectus
INS: insula
